# Revision Q-matrix in middle school chemistry: a structural equation modeling approach

**DOI:** 10.3389/fpsyg.2025.1647968

**Published:** 2025-10-09

**Authors:** Shiwei Lin, Xiaolong Zhang, Guoxu Chen, Xinyu Wang, Yongshuang Ma

**Affiliations:** ^1^Chemistry College, Changchun Normal University, Changchun, Jilin, China; ^2^School of Psychology, Inner Mongolia Normal University, Hohhot, Inner Mongolia, China; ^3^College of Science Education, Changchun Normal University, Changchun, Jilin, China

**Keywords:** Q-matrix, structural equation modeling, middle school chemistry, cognitive diagnosis, revision

## Abstract

The Q-matrix serves as a bridge that links items and attributes, and its accuracy affects the results of cognitive diagnosis. Inaccuracy of the Q-matrix are a common issue frequently encountered in cognitive diagnosis research. This study utilizes the topic “composition and structure of matter” from junior high school chemistry as a representative example, employing Structural Equation Modeling (SEM) to validate the original Q-matrix of the cognitive diagnostic assessment tool. This method prioritizes qualitative analysis results from the perspective of disciplinary connotation, while integrating the signs and significance of regression weights, modification indices (*MI*), and model fit indices, etc. obtained from SEM, to conducted an iterative process of model revision, parameter estimation, and model evaluation, resulting in a better-fitting revised Q-matrix. By employing the generalized deterministic input, noisy “and” gat (GDINA) model, we conducted a comparative analysis between the Q-matrix derived from the SEM approach and those obtained through the following two approaches: the multiple logistic regression-based method utilizing exhaustive search algorithms (MLR-B-ESA), and the multiple logistic regression-based method utilizing priority attribute algorithm (MLR-B-PAA). The findings show that the absolute fit of the Q-matrix derived through the SEM approach had achieved excellent threshold, although it slightly underperformed compared to the benchmark method in terms of comparative data. It is worth noting that the relative fit of the Q-matrix obtained via the SEM approach was superior to that derived from the comparative methods. This suggests that, as the SEM approach emphasizes qualitative analysis grounded in disciplinary connotation, the Q-matrix revision does not strictly conform to the data information obtained from computation. As a result, this may have a certain quantitative impact on the absolute fit. However, in comparative evaluations of methods, the SEM approach exhibits superior performance.

## Introduction

### Cognitive diagnosis

Cognitive diagnosis is a new-generation measurement theory that aligns with the cognitive-level research paradigm (Frederiksen et al., 1993). This approach enables evaluation to move beyond the macro-level of abilities by revealing learners' micro-level cognitive structures, thereby enabling a more nuanced and personalized evaluation of cognitive development and underlying psychological processing mechanisms. This can help reduce the biases introduced by teachers' subjective judgments, thereby facilitating targeted improvements in instruction. As a result, an increasing number of studies are focusing on cognitive diagnosis in the field of education (Tong et al., 2022; Chen, 2024). However, from the perspective of specific academic disciplines, although considerable research has focused on STEM fields (e.g., Jiang et al., 2023; Hu et al., 2021), investigations into the application of cognitive diagnosis in middle school chemistry remain comparatively limited (e.g., Qi et al., 2024; He et al., 2024; Wang et al., 2025).

### Q-matrix

Typically, the Q-matrix is a 0-1 coding matrix with *J* rows (where *J* denotes the number of items) and *K* columns (where *K* denotes the number of attributes), representing the logical correspondences between attributes and items (Chiu et al., 2009; Henson et al., 2009). It specifies the correspondences between items and attributes in cognitive diagnostic assessment tools (Tatsuoka, 1990), and is primarily used to identify the unobservable attributes measured by items and to transform them into observable item response patterns. It links the examinee's unobservable knowledge states with observable item response patterns, thereby providing a foundation for further understanding and inference of the examinee's knowledge states (Tatsuoka, 1990; Song et al., 2024). The incorrect specification of the Q-matrix can be categorized into two types: over-specification and under-specification (Li et al., 2021; Chen et al., 2013). Over-specification refers to the assignment of an excessive number of attributes to an item in the Q-matrix, leading to inaccurate specification in which attributes are erroneously linked to an item that can be correctly answered without requiring mastery of that attribute. In contrast, under-specification represents the precise opposite scenario. Both of them may reduce the accuracy of cognitive diagnosis and result in poor model-data fit (de la Torre, 2008; Im and Corter, 2011; Gao et al., 2017; Liu and Wu, 2023). Therefore, constructing a sound Q-matrix is a critical step in the cognitive diagnosis process (de la Torre and Chiu, 2016; Tatsuoka, 1983). The formation of the Q-matrix is a process defining attributes for items (Luo, 2019) and it mainly encompass two sorts of approaches: qualitative approaches and data-driven approaches.

Qualitative approach is the earliest and most widely employed strategy. Under this methodology, the Q-matrix is established by discipline experts, who assign attributes to pre-designed items based on predefined attribute definitions (Nájera et al., 2020). It includes the following specific practical operational methods such as literature review, theoretical analysis, expert interviews (Nájera et al., 2020) and so on. However, due to cognitive limitations of discipline experts, omissions or errors may occur during the formation of the Q-matrix (DeCarlo, 2011). Therefore, qualitative approaches are relatively subjective (Yu and Cheng, 2020) and are susceptible to introducing errors into the Q-matrix due to researchers' cognitive biases (Chiu, 2013; Li and Suen, 2013).

To enhance the objectivity of the results, researchers have developed a range of data-driven approaches, such as the δ*-*method (de la Torre, 2008), the joint estimation algorithm (Chen and Xin, 2011), the unsupervised and supervised learning schema (Wang, 2012), the Bayesian approach (DeCarlo, 2012), the γ method (Tu et al., 2012), the method based on the Likelihood *D*^2^ statistic (Yu et al., 2015), the non-linear penalized estimation method (Xiang, 2013), the optimization of response distribution purity method (Li et al., 2022), and the method based on the three random forest models (Qin and Guo, 2023). The fundamental principle underlying these methods is that if the attribute definitions of items are inaccurate, the cognitive diagnosis model cannot establish an accurate correspondence with examinees' attribute mastery patterns. This discrepancy results in distortion of the model's functioning and anomalies in its parameter estimates (Rupp and Templin, 2008). Data-driven approaches focus on the fields of statistical algorithms, emphasizing the exploration of algorithmic principles and their mathematical derivations for estimating the Q-matrix. For researchers without relevant professional backgrounds, applying these methods in practical cognitive diagnostic research remains challenging. It is worth noting that, might be due to the inherent complexity of the content in the discipline of chemistry, we haven't seen any reports on the application of data-driven methodologies to Q-matrix revision in cognitive diagnosis within the domain of chemistry. On the other hand, although data-driven approaches can minimize subjectivity in the determination of the Q-matrix and enhance the model's compatibility with the data, they fail to integrate disciplinary relevance into the process of refinement, thereby potentially yielding biased outcomes. This study employs the content of “composition and structure of matter” from junior high school chemistry as a case example, and applies the structural equation modeling (SEM) to address this limitation, efficiently integrating qualitative analysis with data-driven refinement, the complementary strengths of both approaches are effectively combined, thereby enhancing the accuracy of the Q-matrix.

### SEM

SEM is a multivariate statistical method that integrates factor analysis and path analysis (Quintana and Maxwell, 1999) and is used to evaluate the consistency between theoretical hypotheses and empirical data (Fang et al., 2002). The core logic of SEM is to empirically test a predefined theoretical model using observed data and to analyze the causal pathways and complex interaction mechanisms among variables (Moustaki et al., 2004). By utilizing the covariance matrix of variables, the associations among multiple continuous variables can be obtained (Jöreskog, 1988; Kline, 2016). The confirmatory function of SEM is realized by comparing the implied covariance matrix of the theoretical model with the observed covariance matrix derived from empirical data, thereby revealing discrepancies between the theoretical model and empirical observations (Raykov and Marcoulides, 2006; Lee, 2007; Byrne, 2016). This process typically employs fit functions to quantify these discrepancies, with commonly used estimation methods including generalized least squares (GLS; Jöreskog and Goldberger, 1972), maximum likelihood (ML; Jöreskog, 1971), asymptotic distribution free (ADF; Bollen, 1989) and so on.

For Q-matrix, an item often corresponds to multiple attributes, which is consistent with the key feature of SEM that its ability to allow a single observed variable to reflect multiple latent variables simultaneously (Bollen, 2002; Brown, 2006; Muthén and Asparouhov, 2012; Koizumi and In'nami, 2020). SEM can simultaneously handle multiple observed and latent variables (Diamantopoulos and Siguaw, 2000), which aligns well with the structure of the Q-matrix in cognitive diagnosis, as it includes multiple directly observable items and multiple unobservable attributes. From the perspective of validating theoretical models, SEM can compare discrepancies between the covariance matrix implied by the Q-matrix and the covariance matrix derived from actual response data, thereby providing feasibility for revising the Q-matrix. Furthermore, SEM allows for measurement errors in observed variables (Bollen and Long, 1993), which aligns with the reality of guessing and slipping in students' responses. Additionally, multiple well-established software programs options are available for the analysis of SEM (Hair et al., 2013; Byrne, 2016). Based on the aforementioned considerations, this study employs SEM approach to revise the Q-matrix.

### Model hypotheses

**Figure 1:**
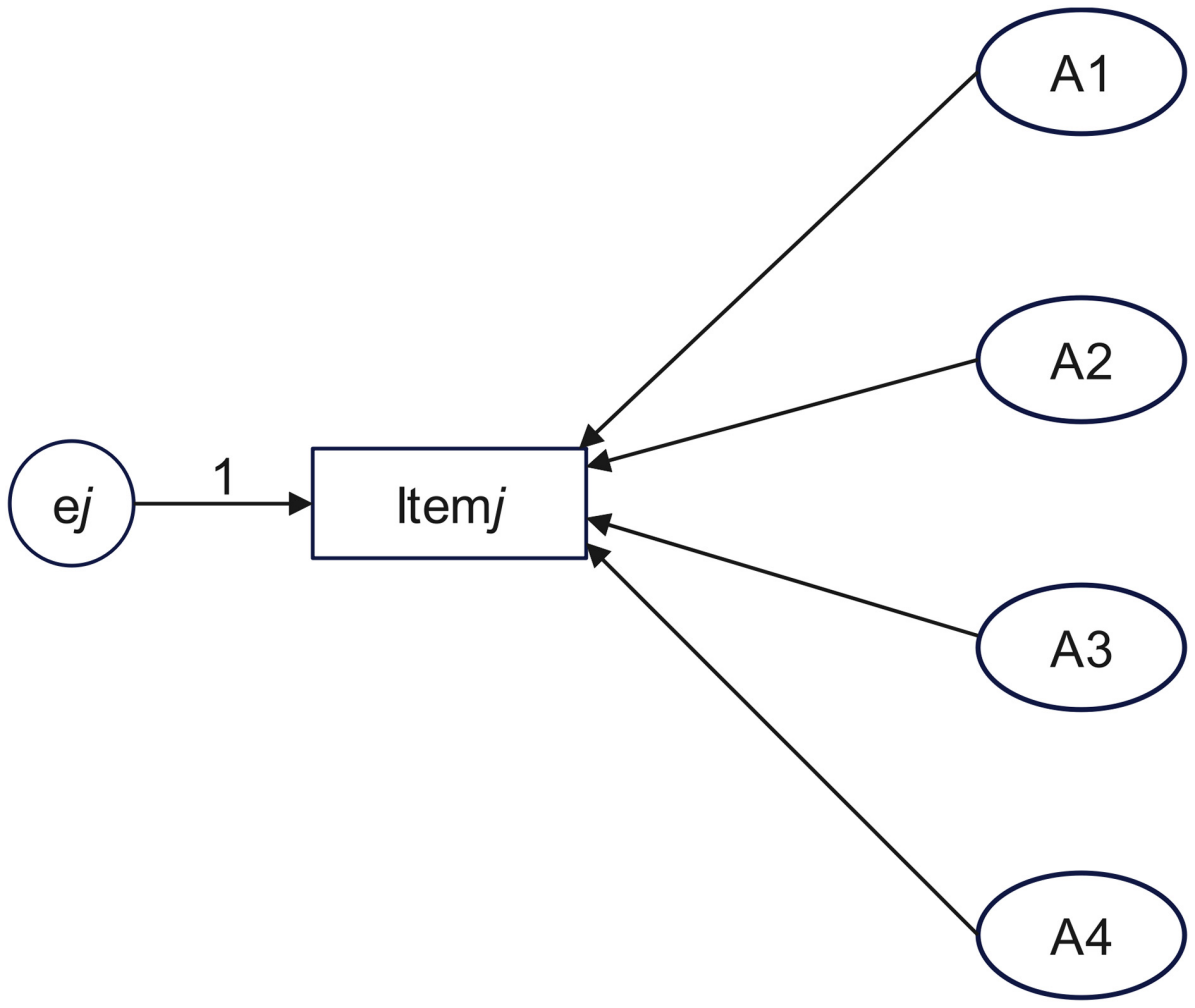
Schematic diagram of establishing paths from attributes to item.

The hypothesized model transforms the frame of Q-matrix into a statistical model by presetting the correspondence paths between items and cognitive attributes, as well as paths between different cognitive attributes. Serving as the foundational starting point for SEM approach research, it provides both the theoretical framework and statistical basis for subsequent data analysis, model fitting, and result interpretation. The hypothesized model comprises two components: the measurement model and the structural model. The measurement model delineates the relationship between observed variables (i.e., items) and latent variables (i.e., cognitive attributes), while the structural model characterizes the relationships among latent variables (e.g., cognitive attributes). Therefore, when applying the SEM approach to revise the Q-matrix, the central objective is the validation and optimization of the measurement model. To enable estimation and validation of the hypothesized model, the subsequent hypotheses are proposed:

**H1** The items are treated as observed variables, and the attributes are treated as latent variables. If an item assesses a specific attribute, then that attribute can, to some extent, account for students' scores on the item. Therefore, a path should be established from the given attribute (latent variable) to the corresponding item (observed variable). Furthermore, the higher a student's level of mastery of an attribute, the greater the probability that they will perform well on related items. There is a significant positive correlation between them. This can be illustrated by the following example:

As shown in Figure 1, assume that Item *j* measures four attributes—A1, A2, A3, and A4—and that each of these attributes can, to some extent, account for the student's score on Item *j*. Four paths should be established: A1 → Item *j*, A2 → Item *j*, A3 → Item *j*, and A4 → Item *j*. It should be hypothesized that all four paths have significantly positive regression weights (*r* > 0, *p* < 0.05), where e*j* denotes the measurement error. If *r* < 0 or *p* ≥ 0.05, this suggests a potential problem in the correspondence between the attribute and the item. Thus, it is necessary to reassess the attributes measured by Item j.

**H2** The following regression weights are assumed to be 1: A1 → Item2, A2 → Item6, A3 → Item13, and A4 → Item1. Fixing the specified regression weights from an observed variable to a latent variable at 1 is a foundational step for defining the latent variable's scale and ensuring model identification. This process effectively establishes a meaningful metric for latent variables by selecting reference indicators, while simultaneously accounting for measurement error, thereby achieving an appropriate balance between theoretical assumptions and empirical data fit. Typically, the observed variable that demonstrates the strongest theoretical linkage and the highest level of reliability is chosen as the reference indicator. Thereby, the criteria for standardizing the regression weights between cognitive attributes and items to 1 are as follows: (1) The item focused exclusively on the examination of a specific attribute, and (2) when multiple items measure the same attribute, the item exhibiting the strongest loading is selected. These criteria were executed through expert consultation and questionnaires to teachers.

**Figure 2:**
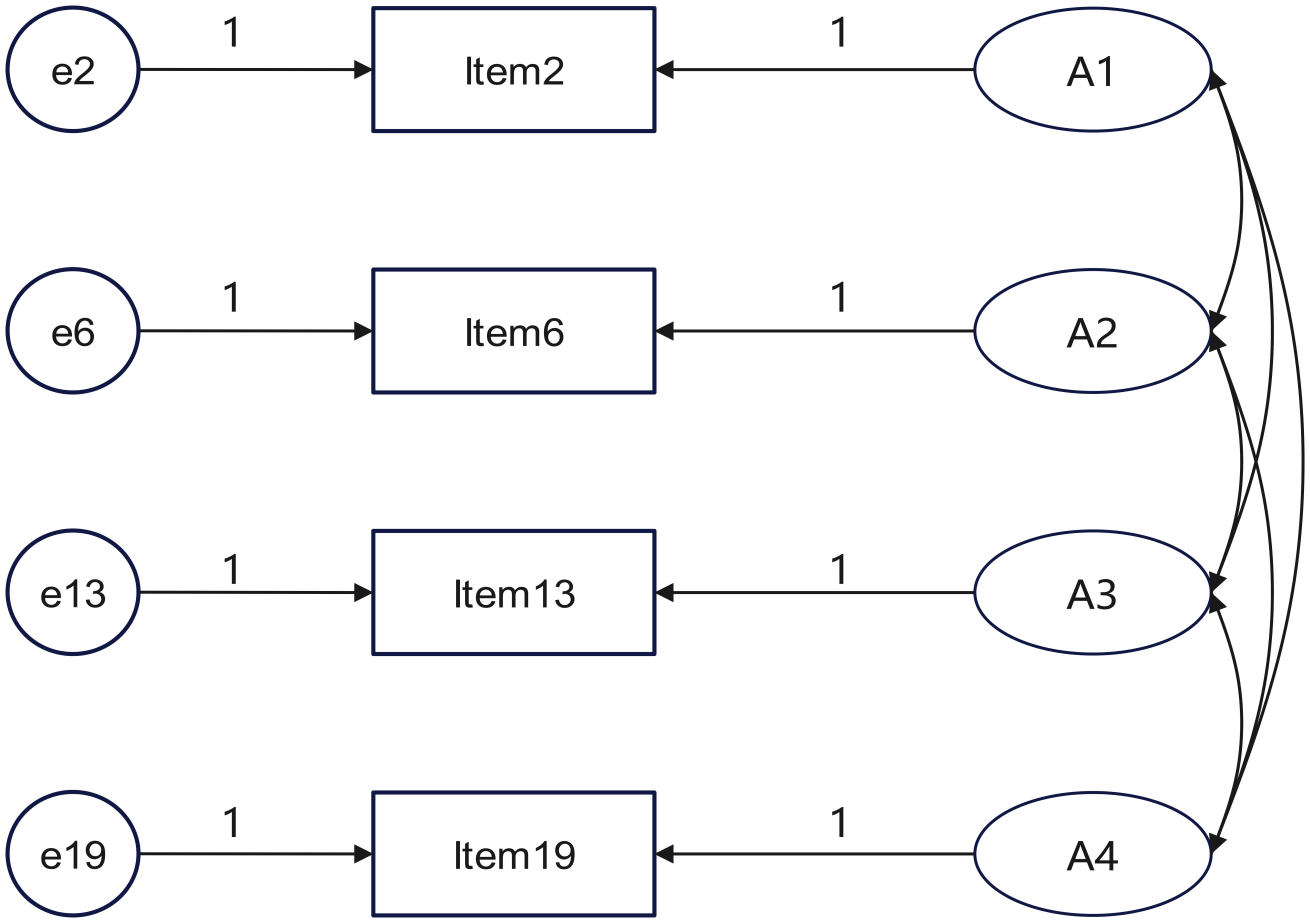
Schematic diagram of fixed path regression weights and covariance relationships between attributes.

**H3** The attributes are not mutually independent but are intrinsically related. Therefore, pairwise covariance among them should be specified in the hypothetical model. Both H2 and H3 can be illustrated by Figure 2.

## Materials and methods

### Study domain

This research focuses on “composition and structure of matter,” one of the five themes outlined in China's chemistry curriculum standards of compulsory education (Ministry of Education of the People's Republic of China, 2022), as its study domain. This theme reveals the composition of matter from a macroscopic perspective and its structure from a microscopic viewpoint, while also providing a theoretical foundation for other themes through the integration of macro- and micro-level perspectives. It serves as an important vehicle for developing students' core literacy in chemistry. According to the content studied, the following cognitive attributes are identified: (A1) foundational conception of “composition and structure of matter;” (A2) representation of “composition and structure of matter;” (A3) foundational principles of “composition and structure of matter;” (A4) investigative methods for “composition and structure of matter.”

### Instruments

A questionnaire survey was administered to discipline experts and junior high school chemistry teachers to evaluate the following aspects: (1) the rationality of the cognitive model for the “composition and structure of matter” content, with particular emphasis on its cognitive attributes; (2) the rationality of the draft of the preliminary Q-matrix. The questionnaire has a five-point Likert scale to assess the extent to which discipline experts and teachers agree with. Furthermore, each item includes an open-ended comment section where discipline experts and teachers can offer specific revision suggestions concerning potential problem.

A cognitive diagnostic assessment tool for “composition and structure of matter” was developed using dichotomous scoring, with 1 point assigned for fully correct responses and 0 points for omissions or incorrect answers. Specially, the multiple-choice items (Item 23, Item 24), and subjective items (Item 30, Item 31) were all dichotomized, with all non-top scores converted to 0. Pilot testing (*N* = 205) led to the elimination of four problematic items, based on the empirical criterion that *RMSEA* > 0.10 indicating poor item quality (Kunina et al., 2009). The final assessment tool comprised 32 items (Furthermore, another 1 item was removed later in the revised Q-matrix obtained by SEM approach), with the frequency of each cognitive attribute was assessed as follows: A1 (19), A2 (17), A3 (13), and A4 (8). The overall *Cronbach's* α of 0.889 indicated good test reliability. Deletion of any item did not lead to an increase in the *Cronbach's* α greater than 0.02 (Bontte, 2002). The actual score of each item showed statistically significant correlations with the total actual score (*p* < 0.01), with correlation coefficients exceeding 0.20 (Gerbing and Anderson, 1988). The correlation coefficient between the predicted total scores derived from the generalized deterministic input, noisy “and” gat (GDINA) model and the students' actual test scores is 0.816. An extreme group analysis was carried out as follows: the total actual scores for each item were ranked in ascending order. The lowest 27% of the scores were categorized as the low-score group, whereas the highest 27% were categorized as the high-score group. The extreme group analysis demonstrated statistically significant differences between the two groups across all items (*t* > 3, *p* < 0.001) (Thissen and Orlando, 2001). The aforementioned analysis demonstrates that the cognitive diagnostic assessment tool exhibits strong reliability and validity.

### Participators

Participators involved in this study, including experts, teachers, and students, are all drawn from Jilin Province, Sichuan Province, and the Guangxi Zhuang Autonomous Region of China. Oral reports were conducted with six representative students from a key middle school in Jilin Province, in order to develop the draft of original Q-matrix. Three of them are high-achieving students (2 male/1 female, mean age 14.3 years, all of whom served as chemistry class representatives) and three are students with average academic performance (1 male/2 female, mean age 14.1 years, two of whom had participated in academic competitions). To establish the draft of original Q-matrix, we also conducted expert interviews. Four experts (2 male/2 female, mean age = 46.3 years, SD = 2.1) were selected, three of whom hold senior professional titles with ≥15 years' middle school teaching experience. All of them are currently involved in, or have previously participated in, municipal-level or higher teaching research projects. In order to evaluate the level of consensus among frontline educators concerning the initial Q-matrix, a questionnaire survey was administered to 26 chemistry teachers (mean age = 39.6 years, SD = 5.2; 57.7% held senior professional titles with ≥10 years' teaching experience; 73.1% female; 57.7% teaching in urban schools and 42.3% in rural settings; 76% currently participating in or have participated in teaching research projects at the municipal level or above). Cognitive diagnostic tests were conducted in samples from six junior high schools in different regions as mentioned above. The locations of these schools include both urban and rural areas, ensuring the sample's representativeness, with a total of 793 students participating. Seven hundred fifty-two (372 male/380 female, mean age 14.3 years) of them provided valid responses, resulting in a valid response rate of 94.8%.

### Data analyses

The statistical results indicate that the surveyed teachers' consistency coefficient was 0.73 (> 0.70), and the variance was 0.20 (< 0.25), suggesting that although teacher agreement with the original Q-matrix falls within an acceptable range, it is near to the threshold, and the rationality of the matrix still requires improvement (Okoli and Pawlowski, 2004; Qxley et al., 2024).

The SEM approach was employed to validate the original Q-matrix using IBM Amos version 28.0 software and the ML was used to parameter estimation. The ordered categorical variables were treated as continuous variables. Hypothetical model was established by the graphical user interface (GUI) of Amos. During the revision process, we have adopted the following strategies: (1) Any dropping or adding of paths should prioritize qualitative analysis based on disciplinary connotation, with data-driven quantitative methods serving only a supplementary role. An iterative process guided by both disciplinary connotation and data derived from SEM was implemented repeatedly. (2) All path coefficients in the hypothesized model were required to satisfy the criteria of *r* > 0 and *p* < 0.05. (3) Modification suggestions derived from SEM are prioritized for implementation in descending order of their modification indices (*MI*) values, because the *MI* values reflect the expected reduction in the model's chi-square statistic upon implementing the suggested change, and higher *MI* value indicates a more substantial improvement in model fit (Saris et al., 1987; Bagozzi and Yi, 1988). (4) Prioritize the establishment of pathways before proceeding to determine covariance. (5) Each time a path or a covariance is added, the model parameters need to be re-estimated. Subsequently, verify whether the *NFI* and *RFI* values have met the threshold, and whether all other parameters remain acceptable (any anomalies indicated a reversal of the modification). Simultaneously, obtain the updated *MI* values and the corresponding suggestions for the subsequent round of modifications. (6) The measurement errors of observed variables are uncorrelated with latent variables (McDonald, 1985; Bollen, 1989; Jöreskog and Sörbom, 1993), therefore no covariances are specified between the measurement errors of observed variables and the latent attributes. (7) No path can be established between observed variables (items) (Kline, 2016; Byrne, 2016). (8) When adopt the suggestions corresponding to the maximum *MI* value resulting in some regression weights statistically insignificant (*p* ≥ 0.05), it indicated that further path additions were no longer effective in improving the model, and thus path specification should be discontinued. (9) The revision process was deemed complete when all model fit indexes criteria were satisfied and all path relationships were consistent with disciplinary connotation. In summary, the SEM approach employs a strategy that integrates both qualitative and quantitative methodologies, prioritizing qualitative analysis due to its alignment with disciplinary connotations. Throughout the iterative process, *MI* values and all modification fit indexes and all adjusted fit indices underwent continuous adjustments, progressively converging toward optimal thresholds.

**Figure 3:**
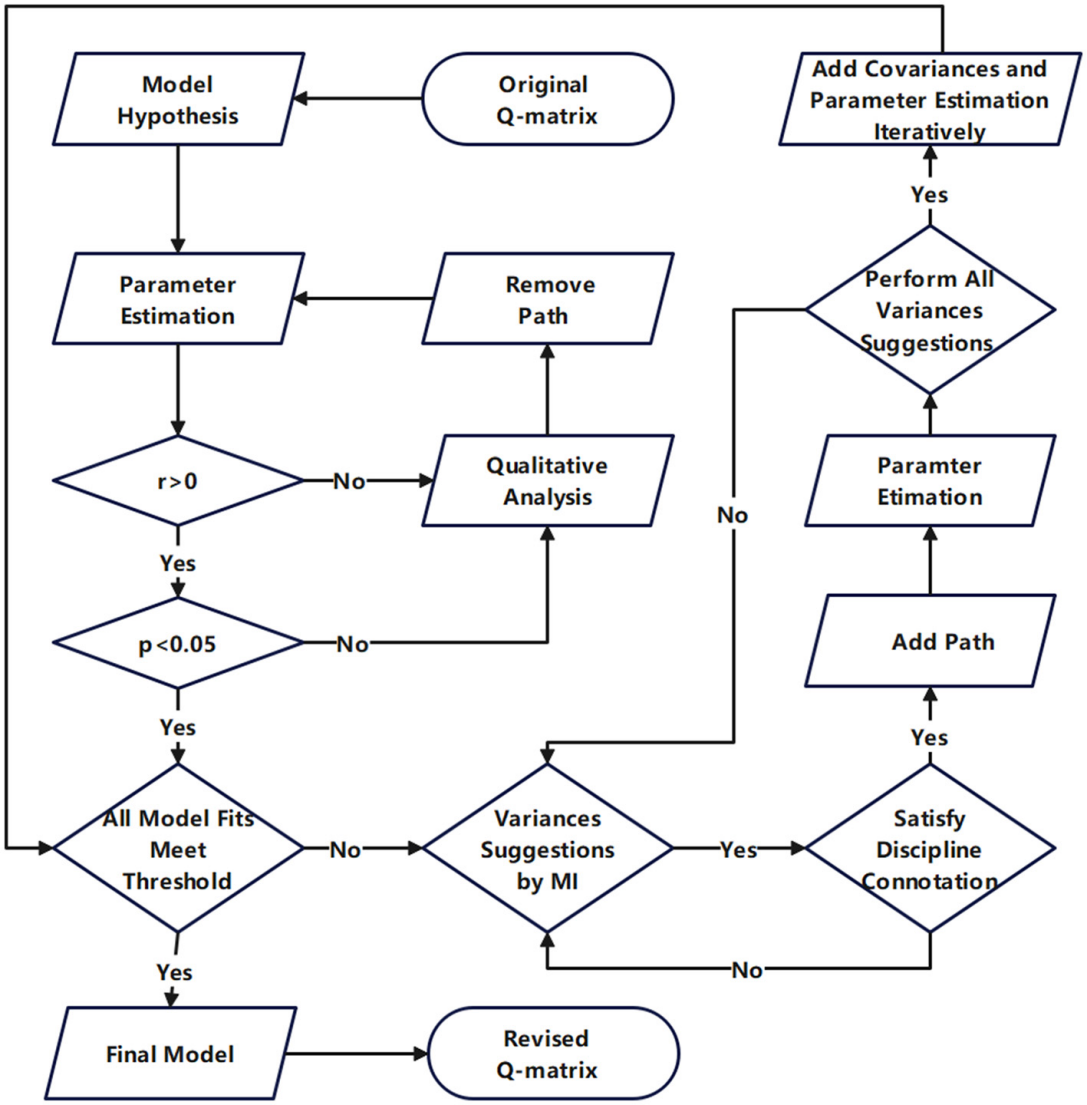
Flowchart for Q-matrix validation by SEM approach.

The overall process is illustrated in Figure 3 and the detailed procedures are outlined as following:

**Step 1:** Establish the hypothesized model in Amos based on the theoretical assumptions and the original Q-matrix. Calculate and estimate the parameters according to the hypothesized model.

**Step 2:** Perform a qualitative analysis of the disciplinary connotation associated with paths exhibiting correlation coefficients *r* < 0, investigate the underlying causes of their irrationality, remove these paths, and subsequently calculate and estimate the parameters.

**Step 3:** Perform a qualitative analysis of the disciplinary connotation associated with paths with *p* > 0.05, investigate the underlying causes of their irrationality, remove these paths, and subsequently calculate and estimate the parameters. At this stage, all path coefficients are expected to meet the criteria of *r* > 0 and *p* < 0.05. If all model fit indexes satisfy the threshold requirements, proceed to Step 5. If any deficiencies are identified, advance to Step 4.

**Step 4:** Examine the suggestions provided by the software for establishing path relationships in descending order of *MI* values. Even if the *MI* value is substantial, the corresponding recommendation will not be adopted if it lacks disciplinary connotation. Calculate and estimate the parameters after adopt a suggestion. Examine whether any anomalies exist in the newly generated path parameters. If anomalies occur, this suggests that the proposed path relationship is statistically or theoretically unjustified and should therefore be rejected. Further qualitative investigation should be conducted to determine the underlying causes of the inconsistency from the perspective of disciplinary connotation. Remove the previously established unreasonable path and recalculate and re-estimate the parameters accordingly. Subsequently, the same approach shall be employed to handle the suggestion with the secondary large *MI* value. Repeat this process iteratively until all the path relationship that aligns with disciplinary connotation and suggestions corresponding to large *MI* values are identified. Through this iterative process, all model fit indices will be further refined. If although the addition of path relationships improves the model fit, concurrently leads to anomalous path parameters, this suggests that the strategy of adding path is no longer effective, and the procedure advances to Step 5.

**Step 5:** Add the covariance relationships among attributes sequentially in descending order of *MI* values, and perform parameter estimation after each covariance relationship addition until all model fit indices reach the desired thresholds. During this process, there will be no newly abnormal path parameters, but only both path parameters and the model fit will be further optimized. In fact, the operation performed in this step does not alter the Q-matrix, but instead functions as an additional validation of the model's rationality. If path parameter anomalies persist after the adding of all covariance relationships, this suggests that certain items within the cognitive diagnostic instrument may be influencing the overall response data structure and it should be eliminated. After the removal of the poorly fitted item, go back to step a and initiate the next round of revisions until both path parameters and model fit indices attain optimal values.

Researchers have proposed a range of approaches for validating the Q-matrix, such as the general discrimination index (GDI) method (de la Torre and Chiu, 2016), the Wald method (Ma and de la Torre, 2020), the Hull method (Nájera et al., 2021), and the multiple logistic regression-based (MLR-B) method (Tu et al., 2022), and so on. Among them the MLR-B approach demonstrates superior performance in both absolute model-data fit and relative model-data fit (Tu et al., 2022). Each of the aforementioned methods can be executed using two iterative strategies: the exhaustive search algorithm (ESA) and the priority attribute algorithm (PAA) (Qin and Guo, 2024). Based on the aforementioned study, in order to validate the robustness of the SEM approach, we conducted a real data comparative study between the revised Q-matrix obtained by SEM approach and the following: (1) the original Q-matrix; (2) the Q-matrix obtained by ESA-based MLR-B method (MLR-B-ESA); and (3) the Q-matrix obtained by the PAA-based MLR-B method (MLR-B-PAA). The data used in the comparison are consistent with those employed in the SEM approach. Given the complexity inherent in scientific domains—particularly in chemistry—only the saturated GDINA model was selected. Parameters were estimated using the ML method and BM algorithm for monotonicity assumption was executed. The MLR-B method was implemented using the Qval R package (Tu et al., 2022), and the model parameters were estimated using GUI of GDINA R package (de La Torre and Akbay, 2019).

## Results

A hypothetical model was specified based on the original Q-matrix, comprising a total of 130 parameters, as presented in Supplementary Table S1. In order to facilitate model identification and support subsequent parameter estimation, the values of 36 parameters were fixed at 1, including four regression weights from attributes to items and 32 regression weights from measurement errors to items. A total of 94 parameters was estimated, including 52 regression weights from attributes to items, 6 covariance among attributes, and 36 error variances for both observed and latent variables. The number of data points amounts to 528, calculated using the formula *k*(*k* + 1)/2, where *k* denotes the number of items included in the hypothetical model, which is 32 in this case. Therefore, the degrees of freedom *df* = 434, which can be obtained by subtracting the number of parameters from the number of data points (528 – 94 = 434). This outcome satisfies the *t*-criterion requirement (*df* > 0), rendering the hypothetical model identifiable and thus estimable (Byrne, 2016; Tabachnick and Fidell, 2007).

In the hypothetical model, the estimated covariances between A1↔ A2, A1↔ A3, A1↔ A4, A2↔ A3, A2↔ A4, and A3↔ A4 were 0.006, 0.007, 0.016, 0.016, 0.016, and 0.022, respectively, all of which were statistically significant (*p* < 0.001). The model fit statistics before and after revisions are presented in Table 1 (Bentler and Bonett, 1980; Hu and Bentler, 1999). For the original hypothetical model, all of the absolute and parsimonious fit indices met the recommended thresholds, and among the incremental fit indices, *IFI, TLI*, and *CFI* satisfied the recommended criteria, whereas *NFI* and *RFI* fell below the acceptable level (< 0.90). These results suggest that although the hypothetical model based on the original Q-matrix generally fits the students' actual response data, the model fit still requires improvement, indicating the revision of the original Q-matrix is necessary.

**Table 1:** The model fit statistics of hypothetical model.

**Statistical measures and their critical values**	**Original**		**Revised**	
	**Estimate results**	**Meets threshold**	**Estimate results**	**Meets threshold**
**Absolute fit indices**				
*RMR* (<0.05)	0.006	Yes	0.006	Yes
*RMSEA* (<0.08)	0.034 (90% CI: 0.031, 0.037)	Yes	0.028 (90% CI: 0.025, 0.031)	Yes
*GFI* (>0.90)	0.932	Yes	0.945	Yes
*AGFI* (>0.90)	0.932	Yes	0.933	Yes
**Incremental fit indices**				
*NFI* (>0.90)	0.873	No	0.922	Yes
*RFI* (>0.90)	0.855	No	0.901	Yes
*IFI* (>0.90)	0.937	Yes	0.958	Yes
*TLI* (>0.90)	0.927	Yes	0.952	Yes
*CFI* (>0.90)	0.936	Yes	0.958	Yes
**Parsimonious fit indices**				
*PGFI* (>0.50)	0.766	Yes	0.781	Yes
*PNFI* (>0.50)	0.764	Yes	0.790	Yes
*PCFI* (>0.50)	0.819	Yes	0.844	Yes
*CN* (>200)	450	Yes	524	Yes
*χ2/df* (<2.00)	1.861	Yes	1.602	Yes

The regression weights from attributes to items of initial hypothetical model corresponding to the original Q-matrix are listed in Supplementary Table S2. According to the theoretical hypothesis, the regression weights for A1 → Item2, A2 → Item6, A3 → Item13, and A4 → Item19 were fixed at 1, and therefore, standard errors (*S.E*.), critical ratios (*C.R*.), and *p*-*values* were not reported for these paths. Two types of discrepancies from theoretical expectations were identified: (1) Negative regression weights (*r* < 0), including A2 → Item3 (*r* = −0.360), A4 → Item3 (*r* = −1.033), A1 → Item9 (*r* = −0.018), A2 → Item14 (*r* = −0.017), A1 → Item18 (*r* = −0.642), A1 → Item20 (*r* = −0.300), A1 → Item21 (*r* = −0.018), and A1 → Item24 (*r* = −0.082). These results suggest that students' mastery of certain attributes is associated with lower scores on the corresponding items, which is in clear contradiction to theoretical expectations. This discrepancy indicates likely errors in the specified relationships between these items and attributes, and the aforementioned paths should therefore be removed. (2) The following path regression weights were found to be statistically insignificant (*p* > 0.05): A1 → Item7 (*p* = 0.119), A1 → Item10 (*p* = 0.228), A1 → Item11 (*p* = 0.863), A1 → Item15 (*p* = 0.089), and A1 → Item23 (*p* = 0.225). These results suggest that students' mastery of attribute A1 does not significantly influence their performance on these items, which is evidently inconsistent with theoretical expectations. This indicates that the paths from attribute A1 to these items should be revised. Subsequently, paths with negative regression weights were iteratively removed from the model. After each removal, model parameters were estimated again to examine whether any anomalies remained. Finally, all paths have *r* > 0 and the regression weights for A1 → Item4 and A3 → Item20 are significant at *p* < 0.01, while those for the remaining paths are significant at *p* < 0.001. No negative values were observed in neither the covariances nor the error variances among the fit statistics. Except for the *NFI* and *RFI*, which did not reach the recommended threshold, all other model fit indices met the criteria. These results suggest that the model's alignment with the students' actual response data has significantly improved; however, there remains room for the further refinement of the original Q-matrix. After the removal of the aforementioned paths, the model modification suggestions generated by SEM for adding either paths or covariance at this stage are presented in Table 2.

**Table 2:** Selected modification suggestions and *MI* values.

**Suggestions**	***MI***	***Par change***
Item5←A2	14.909	0.225
Item12←A2	12.381	−0.234
Item29←Item4	10.546	0.120
Item27←Item4	9.935	−0.102
Item22←A2	7.764	0.173
e4↔e5	16.866	0.017
e26↔e27	16.071	0.015
e29↔A4	15.26	0.006
e3↔e29	12.342	−0.015
e2↔e4	12.121	0.015
e21↔e24	11.888	0.015
e29↔e30	11.709	0.017
e30↔e31	11.516	0.016
e29↔e31	11.183	0.016
e6↔e26	10.400	0.013

Based on the *MI* values and qualitative analysis, paths were added iteratively as recommended. When adding paths according to the suggestions of SEM, if the newly estimated regression weights are not significant, it indicates that the addition of path is unreasonable, and further addition of the paths will no longer be able to improve the fit of the model. Subsequently, six covariances were introduced following *MI*-based recommendations: e2↔ e4, e4↔ e5, e3↔ e29, e26↔ e27, e21↔ e24, and e30↔ e3, ultimately yielding the revised model. The model fit statistics of the revised model are shown in Table 1, and the regression weights from attributes to items of it are listed in Supplementary Table S3.

Throughout the process of Q-matrix revision using SEM, the approach is not purely data-driven. Rather, while data serves as a critical supporting factor, qualitative analysis that is consistent with disciplinary connotations is regarded as the primary basis for decision-making. The following presents an example for each of the three scenarios: dropping an attribute from an item (Sample Item 1), adding an attribute to an item (Sample Item 2), and data assistance enables a more in-depth qualitative analysis (Sample Item 3). The cognitive diagnostic assessment tool used in this study was originally developed in Chinese. All the sample items in this article have been translated into English.

***Sample item 1:*** *(item ID: Item 7; answer: a; single choice question) Scientists have obtained a new type of oxygen molecule (O*_4_*) using ordinary oxygen molecules and charged oxygen ions. Which of the following statements is correct? (a) O*_4_ *is a neutral molecule; (2) One O*_4_ *molecule contains two O*_2_ *molecules; (3) O*_4_ *and O*_2_ *have exactly the same properties; (d) The mixture of O*_4_ *and O*_2_ *forms a pure substance*.

The stem of this item, together with options a, b, and c, employs chemical formulas as a form of chemical representation, thereby exhibiting attribute A2 (representation of “composition and structure of matter”). Option d involves the fundamental concept of “pure substance,” it seems that, from a qualitative analysis perspective, this item also evaluates attribute A1 (foundational conception of “composition and structure of matter”) to a certain extent. Accordingly, in the original Q-matrix, the attribute profile vector for this item is (1,100). However, this is in contradiction to the non-significant results: A1 → Item7 (*p* = 0.120). This prompted us to perform an additional qualitative analysis of the path. In cognitive diagnostic research, it is advised not to assign an excessive number of attributes to a single item (Cai et al., 2013; Peng et al., 2016), as each attribute may encompass a range of content and may exhibit potential hierarchical relationships with others. Over-specification could lead to poor model-data fit. Item 7 simultaneously measures both lower-level content associated with A1 and higher-level content related to A2. However, the contribution of attribute A1 to correctly answering this item is minimal, and the path from A1 to Item 7 should be removed. Employing purely qualitative analysis methods results in inherently ambiguous and highly subjective resolution criteria for this issue. In contrast, the SEM approach offers an exceptionally robust evaluative framework through its path coefficients. The rationale for removing paths such as A1 → Item10, A1 → Item11, A1 → Item15, and A1 → Item23 is analogous to the explanation provided above.

***Sample item 2:*** *(item ID: Item 22; answer: b; single choice question) The number of protons in an atom must be equal to: (a) neutron number; (b) the number of nuclear charges; (c) relative atomic mass; (d) the number of electrons outside the nucleus*.

In the Q-matrices derived from the MLR-B-ESA and MLR-B-PAA approaches, the assessment pattern vectors for this item all are (1,000). However, qualitative analysis based on disciplinary connotations reveals that accurately answering this question requires students to not only understand basic concepts such as neutron number and relative atomic mass but also to be familiar with the following principles: (1) For electrically neutral atoms, the number of positive charges (protons) in the nucleus must equal the number of negative charges (electrons) outside the nucleus; (2) Atoms can gain or lose electrons. Therefore, this item not only assesses basic concepts related to the composition and structure of matter but also tests fundamental principles and rules governing this composition and structure. Through this qualitative analysis, the assessment pattern vector for this item is ultimately determined to be (1,010) when employing the SEM method.

***Sample item 3:****(item ID: Item 1; answer: b; single choice question) Among the following substances, which one is a pure substance? (a) Air; (b) Nitrogen; (c) Petroleum; (d) Milk*.

During the process using the SEM approach, it was observed that the path coefficient *r* for this item is less than 0. This has prompted a more in-depth qualitative analysis of this issue. The content examined in this item pertains to “pure substances” and “mixtures.” From the perspective of knowledge attributes, it belongs to the category of “foundational conception.” At first glance, this appears consistent with Attribute A1 (the foundational conception of “composition and structure of matter”). However, an in-depth analysis of the disciplinary connotations reveals that the concepts under examination are not related to the “composition and structure of matter” In various editions of junior high school chemistry textbooks in China, the concepts of “pure substances” and “mixtures” are introduced at an early stage. At the point when students are first exposed to these concepts, they have not yet begun studying content related to “the composition and structure of matter.” In other words, the understanding of these two concepts does not presuppose prior knowledge of fundamental ideas such as elements, atoms, or molecules. The domain assessed by the cognitive diagnostic assessment tool in this study focuses on “the composition and structure of matter.” The basic concepts within this domain refer to foundational ideas from both a macroscopic perspective, such as elements, and a microscopic perspective, such as atoms and molecules. Therefore, by utilizing the information obtained from SEM as the primary reference and conducting in-depth qualitative analysis from the perspective of disciplinary connotation, item 1 was removed during the revision of the Q-matrix using the SEM approach.

Table 3 shows the adjustments made by the three approaches to the original Q-matrix. The consistency results between the Q-matrices suggested by these approaches and the original Q-matrix are calculated by $\frac{\overset{I}{\underset{i=1}{\sum }}\overset{K}{\underset{k=1}{\sum }}I\left(q_{ik}^{1}=q_{ik}^{2}\right)}{I\times K}$ (where $q_{ik}^{1}$ and $q_{ik}^{2}\;$represent the values of attribute *k* for item *i* in two Q-matrices ***Q***^**1**^ and ***Q***^**2**^, respectively), and they are presented in Table 4. It can be seen that the revised Q-matrix obtained by SEM approach shows high consistency with those obtained by MLR-B-ESA and MLR-B-PAA approaches. Meanwhile, the similarity of Q-matrix obtained by the methods between MLR-B-ESA and MLR-B-PAA is comparatively higher attributed to the fact that both methods are based on the MLR-B framework, differing solely in the search algorithm employed.

**Table 3:** The original Q-matrix and it's revision results.

**Item**	**Item ID**	**A1**	**A2**	**A3**	**A4**
1	Item 2	1^*^	0	0	0^*^
2	Item 3	1^*+^	0^+^	**1**	**1**
3	Item 4	1	0	0	0
4	Item 5	1	**0**	0	0
5	Item 6	0	1	0	0
6	Item 7	**1**	1	0	0
7	Item 8	**1** ^*+^	1	0	0
8	Item 9	**1** ^*+^	1	1	0
9	Item 10	**1** ^*+^	1	0	0
10	Item 11	**1** ^*+^	1	0	0
11	Item 12	**1** ^*+^	0	0	**1**
12	Item 13	0	0	1	0
13	Item 14	**1**	0	1	0
14	Item 15	**1**	1	0	0
15	Item 16	0	0	1	0
16	Item 17	0	1	1	0
17	Item 18	**1** ^*+^	0	1	1
18	Item 19	0	0	0	1
19	Item 20	**1** ^*+^	1	1	1
20	Item 21	**1** ^*+^	0	1	0
21	Item 22	1	**0**	0	0
22	Item 23	**1**	1	0	0
23	Item 24	1^*+^	1	1	0
24	Item 25	**0**	1	0	0
25	Item 26	0	1	1	0
26	Item 27	0	1	1	0
27	Item 28	0	1	1	0
28	Item 29	1^*+^	1	0	0
29	Item 30	0	0	0	1
30	Item 31	0	0	0	1
31	Item 32	0	1	0	1
0	Item 1^#^	1^*^	0	0	0^*^

^#^This item was deleted in the Q matrix revised by SEM. The attributes marked with special symbols imply that some validation methods suggest adjusting them to the opposite elements: bold denotes the suggestion from SEM, ^*^represents the suggestion from MLR-B-ESA, and ^+^indicates the suggestion from MLR-B-PAA.

**Table 4:** Consistency results between Q-matrices.

	**Original**	**SEM**	**MLR-B-ESA**	**MLR-B-PAA**
Original	1.000	0.863	0.903	0.911
SEM	0.863	1.000	0.895	0.903
MLR-B-ESA	0.903	0.895	1.000	0.976
MLR-B-PAA	0.911	0.903	0.976	1.000

As presented in Table 5, SEM suggests the most adjustments to the Q-matrix, proposing modifications to 17 attributes (two 0 → 1, fifteen 1 → 0), while the adjustments made by MLR-B-ESA and MLR-B-PAA approaches are 13 (two 0 → 1, eleven 1 → 0) and 12 (two 0 → 1, ten 1 → 0), respectively. The SEM approach underwent the most extensive modifications, reflecting its greater potential for uncovering the underlying nature of the discipline. The ratios of modification style of 0 → 1 and 1 → 0 are similar for these three approaches (SEM 0.214, MLR-B-ESA 0.182, and MLR-B-PAA 0.2). This phenomenon reveals that over-specification is more prevalent than under-specification in the Q-matrix derived from qualitative analysis. Absolute fit evaluates the correspondence between a specified model and the observed data. In terms of *M*_2_, *RMSEA*_2_, and *SRMSR*, original Q-matrix shows the lowest values, indicating that all the three methods showed no improvement in absolute fitting performance. However, they all remained within the excellent threshold (e.g., all yielded RMSEA < 0.05). Both MLR-B-ESA and MLR-B-PAA yield lower values than SEM. This suggests that although the absolute fit of the SEM approach is excellent, it may not represent the best one. This phenomenon can be attributed to the fact that the SEM approach differs from data-driven approaches, as doesn't rely solely on model modifications suggested by the model-data fit, on the contrary, it prioritizes qualitative analysis aligned with disciplinary connotation. Relative fit is a criterion for comparing the rationality of multiple models. As shown in Table 5, the SEM approach exhibits the lowest −*2LL, AIC, BIC, CAIC*, and *SABIC* values, indicating superior relative fit. This suggests that the SEM approach provides the best explanation of the data and can be regarded as optimal.

**Table 5:** Relative and absolute ft statistics for the Q-matrices.

**Q-matrix**		**npar**	**Relative fit**					**Absolute fit**					**Modifications**	
			***−2LL***	***AIC BIC***		***CAIC***	***SABIC***	***M**_**2**_ **test***			***RMSEA**_**2**_*	***SRMSR***	**0 → 1**	**1 → 0**
								*M_2_*	*df*	*p*				
Original		145	9705	19,701.0	20,371.3	20,516.3	19,910.8	540.8	383	0.000	0.023	0.036	–	–
MLR_B	ESA	113	9707.3	19,640.7	20,163.0	20,276.0	19,804.2	585.7	415	0.000	0.023	0.036	2	11
	PAA	113	9709.0	19,644.1	20,166.5	20,279.5	19,807.6	591.3	415	0.000	0.024	0.036	1	11
SEM		103	9505.0	19,216.0	19,692.2	19,795.2	19,365.1	624.2	393	0.000	0.028	0.042	2	15

“npar” represents the number of parameters. Original represents the original Q-matrix. 0 → 1 and 1 → 0 represent the counts of adjusting 0 to 1 and 1 to 0, respectively.

## Conclusion

The Q-matrix serves as a bridge linking test items to latent attributes and plays a central role in cognitive diagnosis. Its accuracy directly affects the reliability of the diagnostic results. However, due to limitations or biases in understanding the attributes, item content, and their interrelationships, discipline experts may occasionally make errors when constructing the Q-matrix. Although psychometricians have developed a series of data-driven algorithm, these methods often go from one extreme to the other. Due to the absence of expert judgment regarding disciplinary denotation during the process of Q-matrix revision, the outcomes may appear to exhibit favorable parameter estimates, yet they may diverge from the fundamental disciplinary meaning.

In this study, an original Q-matrix was developed through qualitative research firstly. Although expert validation indicated a high degree of consistency, SEM analysis revealed that several model fit indices did not reach optimal thresholds. Subsequently, prioritizing expert qualitative evaluations, a refined Q-matrix was derived through comprehensive integration of data from SEM including the values and significance of regression weights, *MI* values, model fits and other relevant metrics. Comparative analyses against MLR-B-ESA and MLR-B-PAA approaches indicated that the Q-matrix derived through the application of the SEM approach has undergone the most substantial modifications, suggesting its superior ability to capture the underlying structure of the discipline. However, precisely due to this reason, it has somewhat compromised the absolute fit. The absolute fit results indicate that the SEM approach has achieved an excellent fit threshold, although it performs slightly less favorably compared to MLR-B-ESA and MLR-B-PAA in terms of data fit. The relative fit results demonstrate that the SEM approach is the most effective, indicating its superior performance in the comparative analysis of methods. Furthermore, we discover that over-specification is more prevalent than under-specification in the Q-matrix derived from qualitative analysis under the setting of this report.

SEM approach adopted in this study shows the following characteristics: (1) SEM approach exhibits feasibility. SEM is capable of integrating multi-source data, such as items and attributes, to evaluate the Q-matrix. By simultaneously considering the relationships among multiple variables, SEM can uncover latent structures and patterns in the data, thereby more accurately identifying mis-specifications in the Q-matrix. (2) SEM approach exhibits operational convenience. The application of SEM approach does not require a high level of mathematical foundation. The graphical software IBM Amos can offer user-friendly surface to analysis. The operations are straightforward and no coding is required. Therefore, SEM approach demonstrates potential for implementation and promotion within primary and secondary educational settings. (3) SEM approach exhibits excellent accuracy. The SEM approach enables the systematic integration of quantitative data with qualitative insights. This mixed methodology not only addresses the limitations of purely quantitative approaches in terms of disciplinary denotation, but also mitigates the potential subjectivity inherent in purely qualitative analyses, thereby enhancing the accuracy of the Q-matrix. Furthermore, SEM can account for the influence of measurement error on the model by incorporating error terms, thereby enabling a more accurate representation of the relationships among variables.

The SEM approach is applicable in the following scenarios: (1) The user should have extensive familiarity with the disciplinary connotation, preferably being a disciplinary expert or an experienced frontline educator. When applying the SEM approach, it is essential to integrate qualitative analysis with quantitative analysis. Decisions concerning the dropping or adding of paths should not be based solely on parameter estimates, but should also incorporate qualitative reasoning informed by subject-matter knowledge. Consequently, insufficient familiarity with the subject area may lead to the development of an invalid or unreliable Q-matrix. (2) The sample size should be sufficiently large. Generally, a larger sample size contributes to more stable and reliable model estimation results. Conversely, when the sample size is insufficient, it may result in biased parameter estimates and compromised validity of fit indices, which in turn can negatively impact the quality of Q-matrix revision.

## Limitations and future directions

This study has several limitations that warrant our consideration in future research endeavors: (1) Given that the primary focus of this study is methodological development, the sample size was not expanded to a larger scale. Due to the limited sample size, splitting the data could compromise the stability and reliability of the results. Consequently, cross-validation was not performed in this study. Therefore, to obtain more accurate Q-matrix specifications, it is necessary to increase the sample size and undertake a series of in-depth investigations in future research. (2) Given the inherent complexity of chemistry test items, this study focused solely on parameter estimation using the GDINA model within the framework of saturated cognitive diagnostic models. Further investigation is necessary in our future studies to evaluate the accuracy of this methodology under other saturated models and simplified models. (3) We assume that students' mastery of a given attribute exhibits a linear relationship with their scores on the corresponding assessment items. However, actual cognitive processes are highly complex and may exhibit non-linear relationships between attributes and the score of items. This assumption will affect the model's fit with the data, leading to an impact on the accuracy of the estimation results. Therefore, our future research will further explore methodologies for refining the Q-matrix through the application of non-linear SEM (Zhao and Rahardja, 2011).

## Data Availability

The raw data supporting the conclusions of this article will be made available by the authors, without undue reservation.
