# Supplementary material for: Revision Q-matrix in middle school chemistry: a structural equation modeling approach
Source: Front Psychol. 2025 Oct 9;16:1647968. doi: 10.3389/fpsyg.2025.1647968 (PMC12545035; doi:10.3389/fpsyg.2025.1647968)
Supplement: Supplementary file 1 [file Data_Sheet_1.pdf]

Supplementary Material

TABLE S1 | Parameters of hypothetical model.

	Weights	Covariances	Variances	Means	Intercepts	Total
Fixed	36	0	0	0	0	36
Labeled	0	0	0	0	0	0
Unlabeled	52	6	36	0	0	94
Total	88	6	36	0	0	130

TABLE S2 | The regression weights from attributes to items of initial hypothetical model corresponding to original Q-matrix.

	Estimate	*S.E.*	*C.R.*	*P*
Item1← A1	.594	.241	2.468	.014
Item2← A1	1.000			
Item3← A1	9.541	3.702	2.577	.010
Item3← A2	-.602	.230	-2.616	.009
Item3← A4	-1.415	.668	-2.120	.034
Item4← A1	1.217	.352	3.461	***
Item5← A1	1.297	.356	3.645	***
Item6← A2	1.000			
Item7← A1	.452	.290	1.559	.119
Item7← A2	.902	.090	10.070	***
Item8← A1	.606	.305	1.985	.047
Item8← A2	.843	.089	9.506	***
Item9← A1	-.122	.358	-.341	.733
Item9← A2	.436	.088	4.971	***
Item9← A3	.920	.143	6.429	***
Item10← A1	.324	.269	1.206	.228
Item10 ←A2	.798	.084	9.530	***
Item11 ←A1	.044	.255	.173	.863
Item11 ←A2	1.055	.090	11.724	***
Item12 ←A1	3.915	.903	4.334	***
Item13 ←A3	1.000			
Item14 ←A2	-.030	.081	-.375	.707
Item14 ←A3	.917	.119	7.711	***
Item15 ←A1	.507	.298	1.700	.089
Item15 ←A2	1.049	.095	11.054	***
Item16 ←A3	1.253	.123	10.226	***
Item17 ←A2	.965	.091	10.620	***
Item17 ←A3	.641	.103	6.253	***
Item18 ←A1	-4.377	2.287	-1.914	.056
Item18 ←A3	.969	.242	3.999	***
Item18 ←A4	1.476	.416	3.543	***
Item19 ←A4	1.000			
Item20 ←A1	-2.308	2.498	-.924	.355

Item20 ←A2	.521	.135	3.850	***
Item20 ←A3	.574	.216	2.659	.008
Item20 ←A4	1.228	.470	2.610	.009
Item21 ←A1	-.127	.373	-.340	.734
Item21 ←A3	1.031	.150	6.894	***
Item22 ←A1	1.428	.388	3.684	***
Item23 ←A1	.339	.279	1.214	.225
Item23 ←A2	1.091	.094	11.550	***
Item24 ←A1	-.571	.346	-1.652	.098
Item24 ←A2	.988	.094	10.552	***
Item24 ←A3	.768	.126	6.094	***
Item25 ←A2	1.298	.094	13.822	***
Item26 ←A2	.890	.088	10.090	***
Item26 ←A3	.579	.100	5.783	***
Item27 ←A2	.918	.085	10.771	***
Item27 ←A3	.369	.090	4.105	***
Item28 ←A2	.630	.088	7.187	***
Item28 ←A3	.989	.121	8.140	***
Item29 ←A2	1.288	.097	13.346	***
Item30 ←A4	1.268	.087	14.642	***
Item31 ←A4	1.362	.090	15.130	***
Item32 ←A2	.783	.087	8.963	***
Item32 ←A4	.532	.070	7.556	***

S.E., the standard error of estimate; *C.R.*, critical ratio; *p*, *p*-value; ***, *p*-value $<$ 0.001.

TABLE S3 | The regression weights from attributes to items of final model corresponding to revised Q-matrix.

	Estimate	*S.E.*	*C.R.*	*P*
Item2←A1	1.000			
Item3←A1	3.591	1.030	3.487	***
Item4←A1	1.318	.430	3.066	.002
Item5←A1	.706	.340	2.080	.037
Item6←A2	1.000			
Item7←A2	.987	.084	11.751	***
Item8←A2	.951	.083	11.409	***
Item9←A2	.492	.086	5.751	***
Item9←A3	.812	.114	7.113	***
Item10←A2	.866	.078	11.166	***
Item11←A2	1.073	.084	12.843	***
Item12←A1	5.003	1.420	3.524	***
Item13←A3	1.000			
Item14←A3	.913	.104	8.770	***
Item15←A2	1.152	.091	12.724	***
Item16←A3	1.257	.123	10.250	***
Item17←A2	1.014	.093	10.928	***
Item17←A3	.576	.098	5.887	***
Item18←A3	.453	.101	4.462	***
Item18←A4	.701	.072	9.694	***
Item19←A4	1.000			

Item20←A2	.488	.086	5.686	***
Item20←A3	.267	.114	2.348	.019
Item20←A4	.822	.080	10.332	***
Item21←A3	.958	.111	8.609	***
Item22←A1	.988	.405	2.438	.015
Item23←A2	1.166	.090	13.010	***
Item24←A2	1.028	.091	11.261	***
Item24←A3	.519	.096	5.432	***
Item25←A2	1.315	.096	13.652	***
Item26←A2	.913	.090	10.133	***
Item26←A3	.512	.097	5.300	***
Item27←A2	.954	.088	10.845	***
Item27←A3	.273	.087	3.148	.002
Item28←A2	.696	.088	7.902	***
Item28←A3	.906	.116	7.817	***
Item29←A2	1.292	.098	13.157	***
Item30←A4	1.096	.082	13.321	***
Item31←A4	1.189	.085	14.046	***
Item32←A2	.801	.090	8.888	***
Item32←A4	.514	.068	7.571	***
Item5←A2	.347	.066	5.255	***
Item22←A2	.307	.071	4.320	***

S.E., the standard error of estimate; *C.R.*, critical ratio; *p*, *p*-value; ***, *p*-value $<$ 0.001.
